# Investigation on Shear Behavior of Precast Monolithic ECC Composite Beams

**DOI:** 10.3390/ma18133081

**Published:** 2025-06-29

**Authors:** Tingting Lu, Yuxiang Wen, Bin Wang

**Affiliations:** Shaanxi Key Laboratory of Safety and Durability of Concrete Structures, Xijing University, Xi’an 710123, China; 20180173@xijing.edu.cn (T.L.); b635103208@163.com (B.W.)

**Keywords:** composite beams, R/ECC prefabricated shell, prefabricated monolithic structure, shear behavior

## Abstract

This study applied precast engineered cementitious composite (ECC) shells to replace conventional concrete in precast assembled monolithic composite beams to enhance mechanical performance. A new type of precast monolithic ECC composite beam was proposed. Five ECC composite beams and one reinforced concrete (RC) composite beam were designed and fabricated for the experimental study. The failure pattern, failure mechanism, load-bearing capacity, deformability, and stiffness degradation were quantitatively analyzed through the tests. The main findings were as follows: ECC composite beams developed finer and more densely distributed cracks compared to RC composite beams, without significant concrete spalling. The peak load of ECC composite beams was 8.2% higher than that of RC composite beams, while the corresponding displacement at peak load increased by 29.3%. The ECC precast shell delayed crack propagation through the fiber bridging effect. The average load degradation coefficient of the ECC composite beams was 8.2% lower than that of the RC beam. The stiffness degradation curve of ECC composite beams was more gradual than that of RC composite beams, providing an optimization basis for the design of precast beams in structures with high seismic demands. As the shear span ratio increased from 1.5 to 3, the load-bearing capacity decreased by 32.0%. When the stirrup ratio increased from 0.25% to 0.75%, the ultimate load-bearing capacity improved by 28.8%. Furthermore, specimens with higher stirrup ratios showed a 40–50% reduction in stiffness degradation rate, demonstrating that increased stirrup ratio effectively mitigated brittle failure.

## 1. Introduction

In addition to lowering the gravity load and improving the overall seismic performance of structures, the assembled monolithic composite structure combined the benefits of cast-in-place monolithic and prefabricated structures. It also resolved the hoisting issue of a fully prefabricated structure. Assembled monolithic structures are widely used in reinforcement structures, bridge construction, industrial buildings, residential buildings, and other projects [1]. To improve the mechanical performance of RC monolithic frame beams, researchers adopted various methods to enhance their properties [2,3,4].

Engineered cementitious composites (ECCs), through their unique strain-hardening behavior and multiple micro-cracking characteristics, offered a promising approach for enhanced structural performance [5]. Existing research demonstrated that the ECC layer enhanced the shear capacity of beams [6,7], and optimization of the shear-span ratio enabled further utilization of its strain-hardening potential [8]. The U-shaped ECC permanent formwork optimized shear resistance through synergistic interface interaction [9], while ECC material significantly improved the flexural performance of composite beams [10,11,12]. Scholars validated the flexural performance of composite beams and extended parametric analyses using finite element methods [13,14,15]. The core distinction lies in the interface treatment models; cohesive zone models or tie constraints were recommended for UHPC-NC composite beams, whereas the ECC composite beams employed a perfect bond assumption and required the concrete damage plasticity (CDP) constitutive model to capture their multiple cracking behavior. The ECC layer in ECC composite beams enhanced energy absorption capacity by 17.2–24.5% [16]. Research on ECC composite beams reinforced with different fiber types was also extensively documented [17,18,19]. Despite these significant advances, the shear mechanism of ECC composite beams involves multi-scale material-structure coupling effects. Their performance was synergistically influenced by factors such as shear-span ratio, stirrup ratio, and fiber type, necessitating a systematic investigation to support refined design. The coupling effect of shear-span ratio and stirrup ratio on ECC composite beams remained unquantified [20,21]. A damage evolution model based on stiffness degradation and steel reinforcement strain was lacking for ECC composite beams [22,23]; existing shear theories did not incorporate the strain-hardening behavior of ECC for ECC composite beams [24,25].

Previous research predominantly focused on applications where ECC material partially replaced concrete in cast-in-place reinforced concrete structures, served as permanent formwork for RC structures, or was used to strengthen existing RC members, forming composite elements. This study employed ECC material as the outer shell of precast monolithic composite beams. The prefabricated shell, integrated with welded reinforcement mesh, was cast initially and subsequently combined with cast-in-place concrete to form a novel structural member: precast monolithic ECC composite beams with ECC formwork. The experiment was conducted to investigate the influence of key parameters on shear performance. Detailed analyses were conducted on failure patterns, load-bearing capacity, deformation capacity, strength degradation, stiffness degradation, and steel reinforcement strain. The research findings are expected to provide a theoretical basis and support for the engineering application of this structural form.

## 2. Experiment Overview

### 2.1. Specimen Design and Production

**Figure 1:**
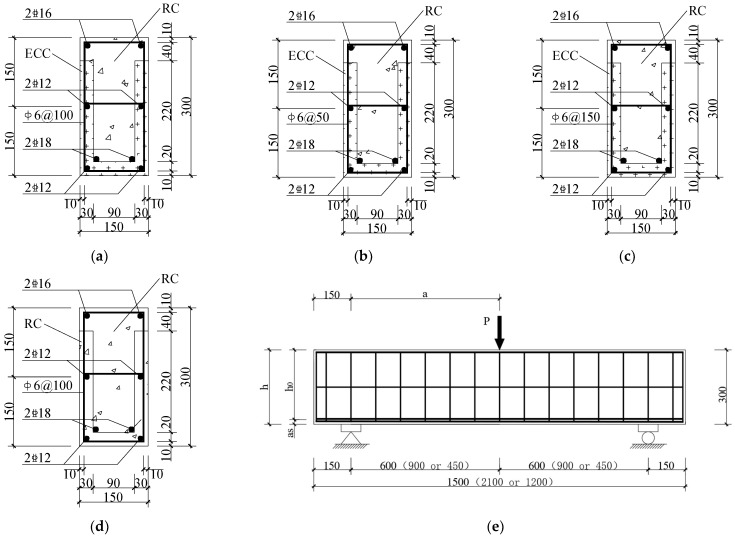
Reinforcement form and section size of the composite beam. (Unit: mm) (**a**) E2S38R; E3S38R; E1.5S38R; (**b**) E2S75R; (**c**) E2S25R; (**d**) R2S38R; (**e**) beam longitudinal section diagram (900 mm and 2100 mm in brackets represent the size of the specimen E3S38R; 450 mm and 1200 mm represent the size of the E1.5S38R specimen).

**Figure 2:**
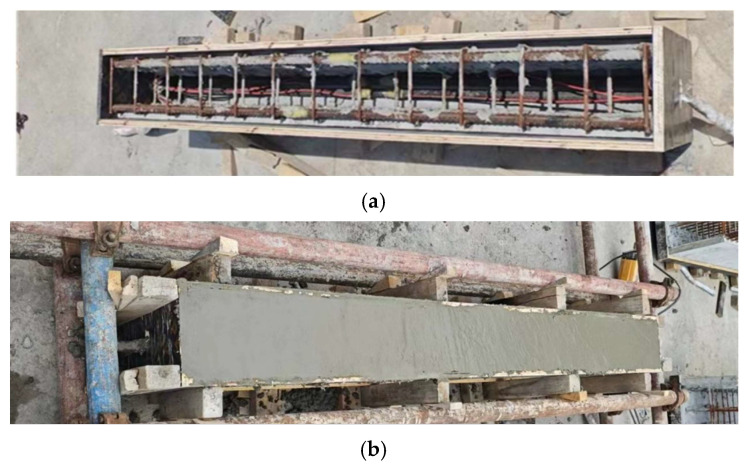
Composite beam pouring: (**a**) the mold shell bottom surface; (**b**) post-pouring concrete.

(1) This study designed five ECC composite beams and one RC composite beam, considering factors such as reinforcement ratio, shear span-to-depth ratio, and different formwork materials. Following the Standard for Test Methods of Concrete Structures [26] (GB/T 50152-2012), the beams were designed as half-scale models. The cross-sectional dimensions of all beams were 150 mm × 300 mm, with a precast formwork thickness of 30 mm. HRB400 steel was used for longitudinal reinforcement, and HPB300 steel was used for stirrups. The primary design parameters of the R/ECC composite beams are summarized in Table 1. The cross-sectional dimensions and reinforcement details are illustrated in Figure 1.

(2) Specimen making: All composite beams in this study were manufactured by a production facility located in Shaanxi Province. Based on the structural configuration and the existing research foundation of our research group, the shell thickness of ECC composite beams was designed to be 30 mm. The concrete pouring procedure for composite beams is depicted in Figure 2. For each batch of R/ECC formwork casting and subsequent concrete pouring, three sets of cubic test specimens, prism test blocks, and dumbbell-shaped tensile specimens were simultaneously prepared. These specimens were utilized to determine the cube compressive strength and axial compressive strength of R/ECC, as well as to measure the stress–strain relationship and uniaxial tensile strength of ECC. All composite beams underwent 28 days of standardized curing under identical environmental conditions.

### 2.2. Material Mechanical

The engineered cementitious composite (ECC) prepared in this study used P.O. 42.5 ordinary Portland cement as the cementitious matrix, and its hydration product, Calcium Silicate Hydrate gel, provided the main bonding strength. Gongyi fly ash was incorporated to optimize the pore structure and inhibit the hydration heat through the pozzolanic effect, forming a dense aggregate system with 0.125–1.18 mm graded quartz sand produced in Shaanxi. The reinforcing phase adopted Japanese Kuraray KURALON K-II PVA fiber (volume content of 2%; various performance indicators are shown in Table 2), utilizing fiber bridging to inhibit crack propagation and improve toughness through the fiber pull-out mechanism. Its high elastic modulus (41 GPa) and hydrophilicity strengthened the fiber–matrix interface bonding. The polycarboxylate superplasticizer improved the fluidity, and the mixture formed an FC40 strength-grade composite material. The material properties are shown in Table 3.

According to the Standard for Test Methods of Concrete Physical and Mechanical Properties [27] (GB/T 50081-2019) and the Standard Test Methods for Mechanical Properties of High Ductility Fiber-Reinforced Cementitious Composites [28], the mechanical properties of the reserved test blocks were evaluated, with the results shown in Table 4.

### 2.3. Test Device and Determination Content

**Figure 3:**
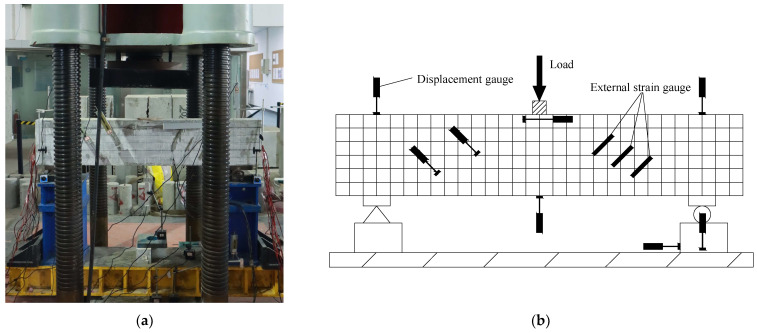
Loading diagram: (**a**) experiment diagram; (**b**) diagram of experimental device.

**Figure 4:**
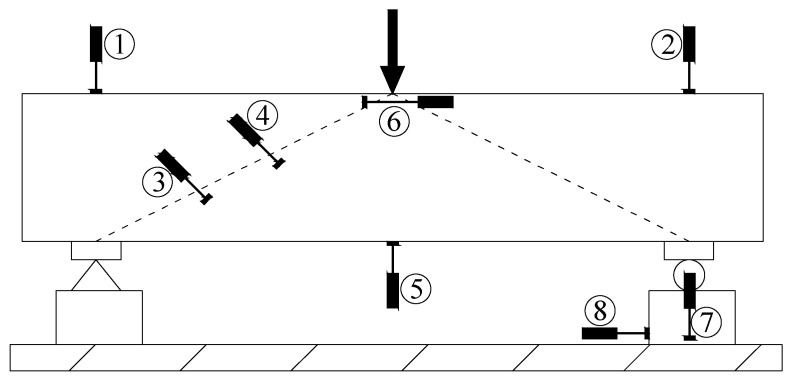
Layout diagram of displacement meter.

**Figure 5:**
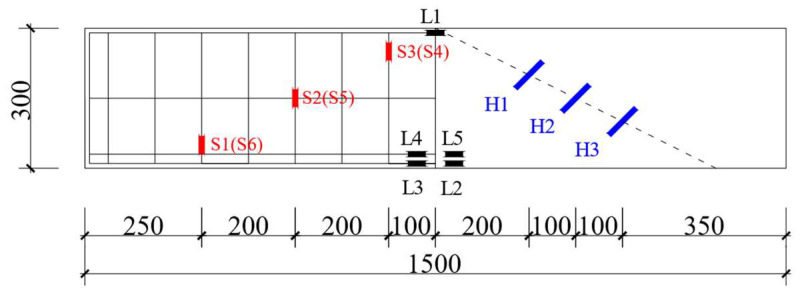
Layout of strain gauges.

(1) Main test equipment: The main test devices used in the shear performance test of the composite beam were the 5000 kN pressure testing machine, Donghua DH3816N static strain test and analysis system, YHD-100 displacement meter, and YHD-50 displacement meter, as shown in Figure 3. The 5000 kN pressure testing machine served as the main test device, and the three-point loading method was adopted. Before the start of the experiment, the geometric center of the test beam was strictly aligned with the loading point. The specimen was preloaded with 10 kN to ensure normal operation of both the test device and measurement equipment, as well as proper contact between the test device and test beam. During the experiment, the pressure sensor of the 5000 kN pressure testing machine recorded real-time test load values. The Donghua DH3816N static strain test and analysis system was primarily connected to the strain gauge channel and displacement meter channel. Displacement meter channel control parameters remain as follows: the measurement type was a bridge sensor, the range was 25,000 mm, the bridge mode was half-bridge, the bridge voltage was 2 V, and the sensitivity was 0.2 mV/mm. Strain gauge channel control parameters are configured as follows: the measurement type is stress and strain, the range is 30,000 με, the bridge voltage is 2 V, and the sensitivity coefficient is 0.2. Before testing, all channels were balanced and zeroed. During testing, real-time values from each channel were collected at a sampling frequency of 2 Hz.

(2) Loading system: In this experiment, the displacement loading method was adopted. The loading rate was set at 0.5 mm/min, with an initial loading displacement of 1 mm per stage and a loading time of 600 s per stage. After reaching 10 mm displacement, the loading increment was reduced to 2 mm per stage, with each stage maintaining a holding time of 600 s. When the displacement reached 30 mm, the loading increment was adjusted to 5 mm per stage, while the holding time for each stage remained at 600 s. The test was terminated when the displacement reached 600 mm.

(3) Displacement measurement: In this experiment, the displacement meter (Shanghuang, Liyang City) was used to measure ranges of 100 mm and 50 mm. A total of 8 displacement meters were set up, and the displacement meter layout is shown in Figure 4. Among them, displacement meters No. 1 and No. 2 were used to monitor the displacement of the loading point. No. 3 and No. 4 displacement meters primarily recorded the surface displacement in the vertical direction of the beam loading point and the bearing connection. Before the experiment, the distance between the two supporting points of No. 3 and No. 4 displacement meters was manually recorded. Combined with the displacement meter data, the concrete strain was calculated, and the calculated data served as a supplement to the concrete strain gauge data. The displacement of the mid-span bottom surface of the beam was recorded by the No. 5 displacement meter, and the load–displacement curve was plotted from the load values recorded by the pressure testing machine for test result analysis. The No. 6 displacement meter was used to monitor the lateral displacement of the upper beam. The displacement data recorded by No. 7 and No. 8 displacement meters were used to determine whether lateral displacement or settlement of the bearing occurred during the test.

(4) Crack measurement: In the preparation process of the test, the white putty paste was brushed on the surface of the test beam, and a square grid of 50mm × 50mm was drawn on the front and back of the test beam, which facilitated later crack measurement and observation. The crack width was measured by the KON-FK (B) crack width monitoring instrument (KONCRETE, Beijing, China). During the loading period after the end of each load stage in the test, the cracks were traced with red pencils, and the width and length of the cracks were measured and recorded in detail.

(5) Strain measurement: The strain gauge was wrapped with epoxy resin on the steel bar and pasted on the concrete surface with glue to obtain the strain changes of the steel bar and concrete during the test. The models of the strain gauges used in this experiment were a longitudinal reinforcement strain gauge (BX120-3AA), a stirrup strain gauge (BX120-3AA), and a concrete strain gauge (BX120-80AA). The electrical characteristic parameters of the strain gauges included a nominal resistance of 120 ± 0.5 Ω and a sensitivity coefficient of 2.11 ± 0.01. The engineering unit was microstrain (με). The specific strain gauge layout is shown in Figure 5, where S represents the stirrup strain gauge, L represents the longitudinal reinforcement strain gauge, and H represents the concrete strain gauge.

## 3. Experimental Results and Analysis

### 3.1. Experimental Phenomena

In this test, the specimens were placed on a 5000 kN pressure testing machine. The front of the beam served as the main observation surface, while the concrete strain gauge, displacement meter, and other instruments were installed on the back of the beam. The following section describes the experimental phenomena and failure patterns of each specimen. No detachment or slippage was observed at the interface between the concrete at the beam top and the ECC formwork, indicating that bond failure did not occur. This demonstrated the excellent integrity of the R/ECC composite beam.

**Figure 6:**
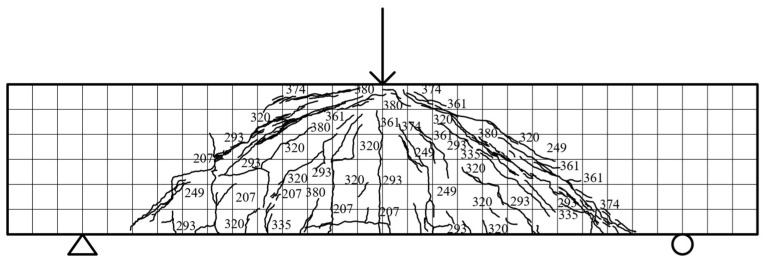
E2S38R failure fracture distribution.

Specimen beam E2S38R: The specimen exhibited initial vertical cracking (width: 0.05 mm, length: 153 mm) at the mid-span back surface under 121 kN load, accompanied by a displacement of 4.02 mm. As the loading increased to 374 kN, diagonal shear cracks propagated toward the loading points, with post-cast RC concrete initiating localized crushing and vertical crack widening to 3.2 mm. The ultimate failure occurred at 383.97 kN (displacement: 17.48 mm), characterized by interconnected diagonal shear cracks (maximum width: 2.2 mm) and vertical flexural cracks extending to five-sixths of the beam height. Post-peak behavior showed progressive concrete spalling at loading points, with main diagonal cracks coalescing into 9.2 mm-wide fractures at 22 mm displacement. The failure pattern combined shear-dominated diagonal cracking and flexural–shear interaction, as illustrated in Figure 6.

**Figure 7:**
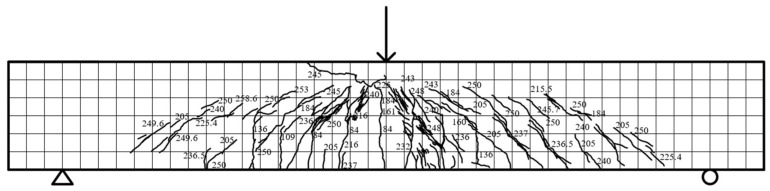
E3S38R fracture distribution.

Specimen beam E3S38R: The specimen exhibited linear elastic behavior with negligible stiffness degradation until initial cracking occurred at 83.54 kN, marked by three vertical cracks (148–152 mm) in the mid-span and left regions. As loading increased to 160.8 kN, vertical cracks propagated to half the beam height, while oblique microcracks (60–193 mm) emerged near the mid-span and supports. Upon reaching 225.4 kN, vertical cracks widened to 1.02 mm, and a diagonal crack initiated coalescence, accompanied by compressive crushing of concrete at 236.5 kN. Peak load (260.1 kN, displacement 48.64 mm) triggered five dense diagonal cracks (63.4 mm average length) near the loading point and vertical cracks exceeding 5.1 mm width. Failure manifested as interconnected diagonal cracking and concrete crushing, with crack distribution illustrated in Figure 7.

**Figure 8:**
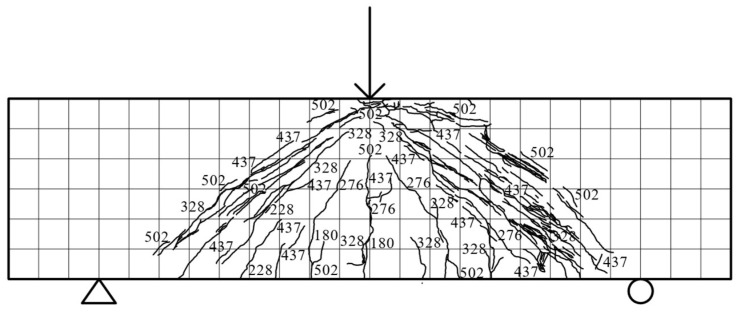
E1.5S38R fracture distribution.

Specimen beam E1.5S38R: The load–displacement curve exhibited linear behavior in the elastic stage, with no stiffness degradation before cracking. Initial vertical microcracks (98 mm and 91 mm) emerged in the front-span tensile zone at 175.9 kN, followed by oblique microcracks developing symmetrically near the mid-span at 227.8 kN. As loading increased to 275.9 kN, vertical cracks propagated to half of the beam height, while dense oblique microcracks formed between supports and loading points, ultimately reaching five-sixths of the section height. At 502.3 kN, vertical cracks widened to 0.27 mm, oblique cracks extended to loading points, and compressive zone concrete crushed, coinciding with the ultimate load of 507.92 kN and a mid-span displacement of 14.62 mm. Post-peak, the bearing capacity declined to 50% at 20.02 mm displacement, with failure characterized by combined shear–flexural cracking and concrete crushing, as illustrated in Figure 8.

**Figure 9:**
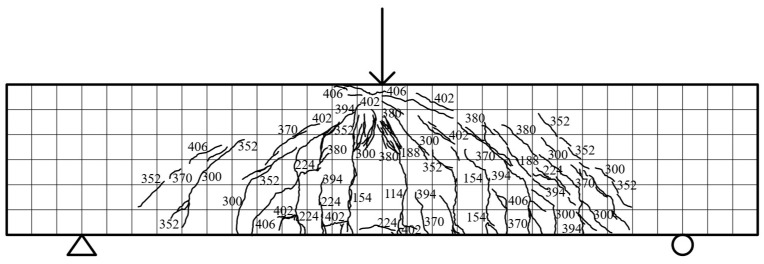
E2S75R fracture distribution.

Specimen beam E2S75R: The load–displacement curve of specimen E2S75R remained linear in the elastic stage (≤114 kN) without stiffness degradation. At 188 kN, inclined microcracks (181.5 mm long, 0.01 mm wide) initiated at the mid-span bottom, while a vertical crack (149 mm long) formed 76 mm from the mid-span on the left rear side. By 300 kN, 28 discontinuous diagonal cracks propagated to five-sixths of the beam height (ECC-RC composite interface) along the front right support-loading line. The peak load of 444.07 kN coincided with 81 diagonal cracks and a 2.6 mm-wide mid-span vertical crack. Failure involved concrete crushing (380 kN) and a 60% post-peak capacity drop (42.4 mm displacement), with crack patterns illustrated in Figure 9.

**Figure 10:**
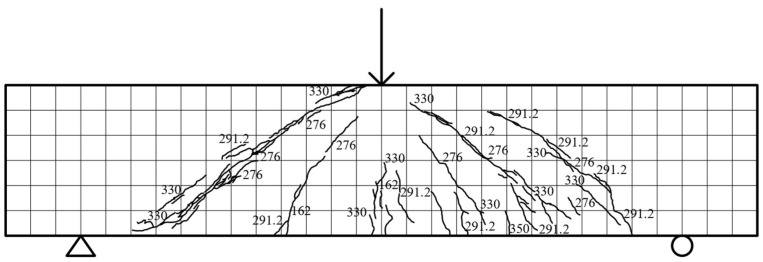
E2S25R fracture distribution.

Specimen beam E2S25R: Specimen E2S25R exhibited a linear load–displacement relationship in the elastic stage (≤162 kN) without stiffness degradation. At 162 kN, vertical cracks (83.5 mm and 51.3 mm long) initiated at the mid-span and 148.3 mm left of the beam bottom, accompanied by a 136.8 mm-long crack on the rear side. Diagonal microcracks first emerged near the support-loading points at 276 kN, propagating to five-sixths of the beam height (ECC-RC interface), totaling 31 cracks, while the rear developed 6 diagonal cracks (max. 162.1 mm). By 330 kN, the front diagonal cracks increased to 58 with 0.11 mm maximum width, and the rear cracks reached 35 (max. 296.8 mm). The ultimate load of 344.7 kN (10.33 mm displacement) preceded concrete crushing near the loading point and diagonal crack penetration (4.2 mm width), with failure patterns illustrated in Figure 10.

**Figure 11:**
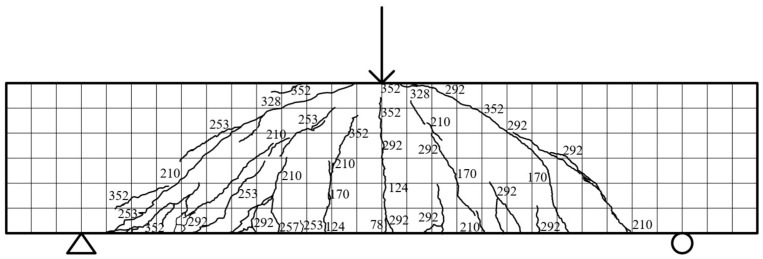
R2S38R fracture distribution.

Specimen beam R2S38R: Specimen R2S38R demonstrated a linear load–displacement response in the elastic stage (≤78 kN) without stiffness reduction. At 78 kN, the first vertical crack (102 mm long) emerged 9 mm right of the mid-span, marking the loss of tensile capacity in the concrete. By 170 kN, the second vertical crack extended 756 mm, while two diagonal cracks (max. 233.6 mm long, 0.03 mm wide) initiated on the right side, accompanied by a 112.6 mm-long vertical crack at the rear mid-span. The crack count increased to seventeen (including six diagonal cracks reaching 397.7 mm) at 253 kN, with rear diagonal cracks propagating to two-thirds of the beam height (max. width 0.13 mm). The ultimate load of 352 kN (12.36 mm displacement) coincided with the interconnection of six primary cracks and diagonal crack widening (max. 0.37 mm rear width), as depicted in Figure 11.

### 3.2. Bearing Capacity Analysis

Through the test records, the cracking load, yield load, and ultimate load of each specimen are shown in Table 5.

(1) As presented in Table 5, specimen E2S38R demonstrated significant performance improvements compared with specimen R2S38R when ordinary concrete was replaced with engineered cementitious composite (ECC). The experimental results revealed a 3.4% increase in yield load, an 8.2% enhancement in ultimate bearing capacity, and a substantial 29.3% improvement in displacement corresponding to the ultimate load. Both composite beams maintained identical geometrical and reinforcement parameters, including shear span ratio, reinforcement configuration, and stirrup ratio, except for their shell materials. The enhanced mechanical performance of the ECC-incorporated prefabricated shell composite beam was primarily attributed to the superior material properties of engineered cementitious composite (ECC). The observed increases in yield load and bearing capacity directly correlated with ECC’s exceptional strength characteristics, while the remarkable improvement in displacement capacity reflected the material’s enhanced ductility and durability. These material advantages effectively improved the structural integrity and deformation capacity of the composite system under loading.

(2) In addition to the different shear span ratios of specimens E1.5S38R, E2S38R, and E3S38R, the other parameters, such as shell material, reinforcement form, and stirrup ratio, were the same. As shown in Table 5, the shear-bearing capacity of the specimens decreased with the increase in the shear span ratio. When the shear span ratios were 1.5, 2.0, and 3.0, the ultimate shear bearing capacity of the specimens decreased by 24.4% and 32%, respectively, while the yield load decreased by 28.7% and 53.2%, respectively.

(3) Except for the different stirrup ratios, the other parameters, such as shell material, reinforcement form, and shear span ratio of specimens E2S25R, E2S38R, and E2S75R, were the same. By comparing and analyzing Table 5, it was concluded that the bearing capacity of specimens increased with the increase in the stirrup ratio, and the stirrup ratios of E2S25R, E2S38R, and E2S75R were 0.25%, 0.38%, and 0.75%, respectively. The yield load of the specimens increased by 26.1% and 9.6%, respectively, and the shear-bearing capacity increased by 10.23% and 13.53%, respectively.

The load-bearing capacity test values of each specimen were compared with the values calculated according to Specification [29]. The calculated load-bearing capacities of each ECC specimen according to the design code were 255.3 kN, 170.2 kN, 340.5 kN, 255.3 kN, and 255.3 kN, respectively. The ECC composite specimens showed bearing capacities consistently higher than the code-predicted values. The primary reason is that the code-specified calculation method is based on elastic theory, whereas the specimens progressively entered nonlinear stages during loading. Thus, the actual capacity exceeds code values. The code provisions were based on conventional concrete members. This study used ECC material, which has different mechanical properties. The ECC’s mechanical properties and material nonlinearity should be considered to establish the shear capacity calculation formula for ECC precast shell assembled monolithic composite beams.

### 3.3. Deformation Analysis

**Figure 12:**
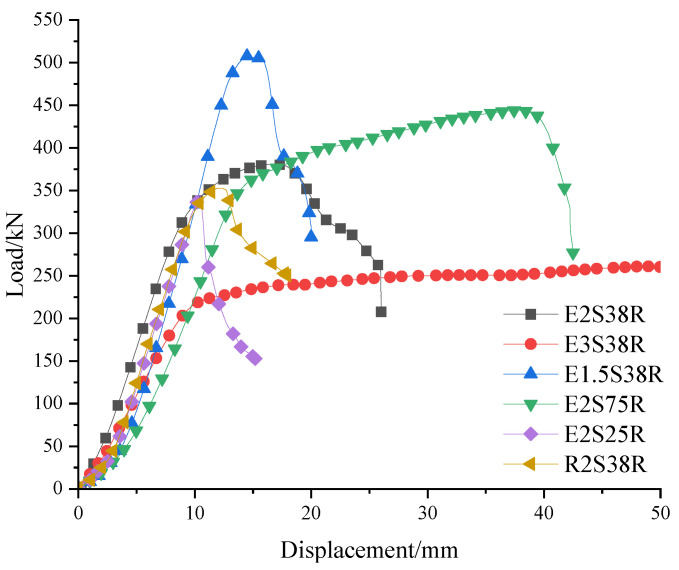
Load–mid-span deflection curve of test beams.

The load–displacement relationships of all specimens were established through experimental measurements, as illustrated in Figure 12. Analysis of these curves enabled systematic evaluation of stiffness evolution and ductility characteristics. Except for specimen E3S38R, all test specimens exhibited three distinct deformation phases: (1) linear elastic stage, (2) elastoplastic loading stage, and (3) post-peak softening phase. During the initial elastic stage, all specimens demonstrated proportional linear responses with minimal stiffness degradation, as indicated by nearly identical slopes in their load–displacement curves before concrete cracking. Transition to the elastoplastic stage coincided with extensive crack network formation, marking the commencement of cracked section behavior. This phase exhibited progressive stiffness reduction correlated with sequential yielding of longitudinal reinforcement and stirrups, accompanied by significant nonlinear deformation accumulation. The specimens ultimately reached their peak load capacities through the development of critical diagonal shear cracks, followed by abrupt shear-dominated failure patterns. This failure mechanism manifested as a rapid load-bearing capacity reduction in the descending branch of the curves, characterized by substantial post-yield displacement accumulation before structural collapse.

(1) Except for the different materials of the mold shell, the other parameters, shear span ratio, reinforcement form, and the stirrup ratio of specimens E2S38R and R2S38R were the same. As shown in Figure 12, under the ultimate bearing capacity, the corresponding displacement of specimen E2S38R was 17.48 mm, while that of R2S38R was 12.36 mm. The displacement corresponding to the ultimate load of E2S38R was 29.3% higher than that of R2S38R. Similarly, the displacement of E2S38R under yield load was 5.4% higher than that of R2S38R.

(2) Specimens E3S38R, E2S38R, and E1.5S38R formed the shear span ratio comparison group. Their shell material, stirrup ratio, and cross-sectional form were identical, with shear span ratios of 3.0, 2.0, and 1.5, respectively. Figure 12 shows that in the elastic stage, the initial slopes of the load–displacement curves differed due to varying specimen lengths (*L*_0_ = 1800 mm, 1200 mm, and 900 mm, respectively). The initial slope increased as L_0_ decreased. With shear span ratios of 3.0, 2.0, and 1.5, the displacement at yield load increased as the shear span ratio decreased, with growth rates of 38.4% and 16.2%, respectively. Conversely, the displacement at ultimate load decreased as the shear span ratio reduced, with reductions of 64.0% and 16.4%, respectively.

(3) For specimens E2S25R, E2S38R, and E2S75R, all parameters—including shell material, reinforcement form, and shear span ratio—were identical except for the stirrup ratio, which was 0.25%, 0.38%, and 0.75%, respectively. Figure 12 indicates that as the stirrup ratio increased, the ultimate bearing capacity grew by 10.23% and 13.53%, respectively. The peak load displacements were 10.33 mm, 17.48 mm, and 37.66 mm, corresponding to growth rates of 40.9% and 53.4%. The yield load displacements were 8.42 mm, 11.00 mm, and 20.51 mm, with increases of 23.5% and 46.4%, respectively.

### 3.4. Bearing Capacity Degradation Analysis

When the specimen reached the peak load, with the increase in load, the plastic damage inside the specimen accumulated continuously, which led to the degradation of the bearing capacity of the specimen. In this study, the bearing capacity degradation coefficient *λi* was introduced to analyze the damage accumulation degree of the specimen. The specific calculation formula is shown in (1).

(1) $$\lambda_{i}=\frac{\left|P_{i-1,max}-P_{i,max}\right|}{P_{i-1,max}}$$

In the formula, *P_i_* is the peak load of the *i*-level loading, and *P_i_*_−1_ is the peak load of the *i*-level loading.

**Figure 13:**
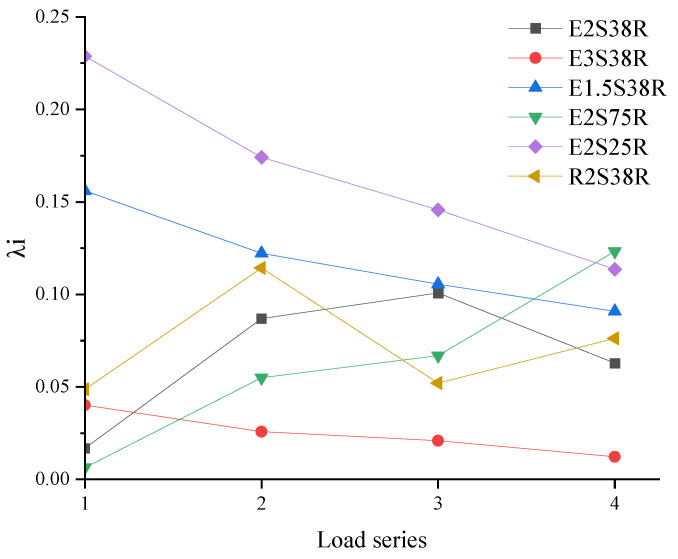
Degradation curve of bearing capacity.

The bearing capacity degradation coefficient calculated according to Formula (1) is shown in Table 6, and the curve drawn by the bearing capacity degradation coefficient and the load series is shown in Figure 13. Among them, because specimen E3S38R was a flexural failure, there was no obvious bearing capacity degradation in its load–displacement curve. Therefore, the calculated bearing capacity change rate of the E3S38R specimen (calculated from 225.72 kN) and the curve drawn by the load series were also placed as a reference in the figure, which was convenient for comparative analysis.

It could be seen from Figure 13:

(1) Comparative analysis of mold shell materials (E2S38R vs. R2S38R) revealed distinct post-peak bearing capacity degradation patterns. As shown in Figure 13, specimen R2S38R exhibited accelerated load-bearing deterioration following peak load attainment, particularly under first-cycle (0.049 vs. 0.017, +65.31%) and second-cycle loading conditions (+23.68%). In contrast, specimen E2S38R demonstrated more pronounced degradation during subsequent loading cycles, with third-cycle degradation coefficients exceeding R2S38R’s values by 48.51% (0.101 vs. 0.052). The four-cycle averaged bearing capacity degradation coefficient for E2S38R (0.067) measured 8.22% lower than that of R2S38R (0.073), statistically confirming the ECC shell material’s capacity to mitigate degradation rates. This phenomenon originated from ECC’s unique strain-hardening behavior, which delayed abrupt post-peak capacity reduction through distributed microcracking mechanisms. The delayed degradation profile of E2S38R particularly manifested during early post-peak loading phases, where fiber bridging effects effectively maintained structural integrity beyond yield points.

(2) The shear span ratios of specimens E3S38R, E2S38R, and E1.5S38R were 3, 2, and 1.5, respectively. The average value of the bearing capacity degradation coefficient of specimen E1.5S38R (with the smallest shear span ratio) was 0.118. Under the first-stage load, the bearing capacity degradation coefficient of specimen E1.5S38R (0.156) was 89.1% higher than that of specimen E2S38R (0.017), which indicated that specimens with smaller shear span ratios were more prone to initial damage due to local stress concentration. Under the second-level load, the bearing capacity degradation coefficient of specimen E1.5S38R (0.122) was 28.7% higher than that of specimen E2S38R (0.087), demonstrating that damage propagation accelerated more rapidly in specimens with smaller shear span ratios as the load increased. Under the third and fourth-level loads, the load degradation coefficients of specimen E1.5S38R remained higher than those of specimen E2S38R. These results confirmed that smaller shear span ratios corresponded to more significant bearing capacity degradation.

(3) Comparing different stirrup ratios, the stirrup ratios of specimens E2S25R, E2S38R, and E2S75R were 0.25%, 0.38%, and 0.75%, respectively. The stirrup ratio of specimen E2S25R was the lowest, which led to the rapid decrease in bearing capacity after peak load. At the first stage load, the bearing capacity degradation coefficient of specimen E2S25R (0.229) was 92.58% and 97.38% higher than that of specimens E2S38R (0.017) and E2S75R (0.006), respectively. After the second and third loads, the bearing capacity degradation coefficient of specimen E2S25R was also greater than that of specimens E2S38R and E2S75R. Due to the high stirrup ratio of specimen E2S75R, the degradation of the bearing capacity of the specimen after the peak load was not obvious, so the degradation coefficient of the bearing capacity of specimen E2S75R was less than that of specimens E2S38R and E2S25R in the first three loads. When loading to a large displacement, that is, under the fourth load, the bearing capacity of specimen E2S75R had a significant decrease, so that the degradation coefficient of its bearing capacity was higher than that of specimens E2S38R and E2S25R. The average degradation coefficients of bearing capacity of specimens E2S25R, E2S38R, and E2S75R were 0.166, 0.067, and 0.063, respectively. It showed that the average value of the bearing capacity degradation coefficient of the specimen decreased with the increase in the stirrup ratio. The E2S25R specimen exhibited brittle failure due to the low stirrup ratio, and the bearing capacity degradation coefficient was relatively large.

### 3.5. Stiffness Degradation Analysis

Under sustained load, the stiffness of the specimen decreased with the increase in displacement. The stiffness variation of the specimen was analyzed by secant stiffness *K_i_*. The calculation formula is shown in (2):

(2) $$K_{i}=\frac{F_{i}}{U_{i}}$$

In the formula, *K_i_* is the secant stiffness of the specimen under the *i*-level loading; *F_i_* is the peak load of the *i* load; *U_i_* is the displacement corresponding to the peak load during the *i*-stage loading.

**Figure 14:**
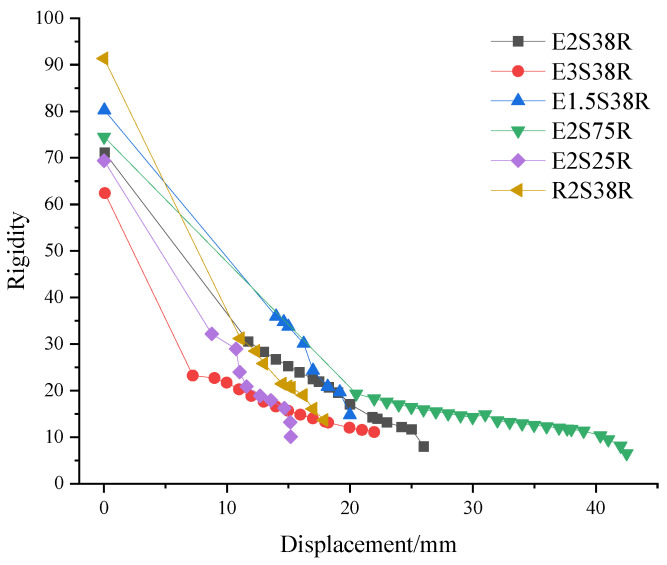
Stiffness–displacement curve.

The stiffness displacement curve calculated by Formula (2) is shown in Figure 14.

(1) As the applied load increased, cracks and internal damage within the specimen progressively accumulated, while the specimen stiffness exhibited a continuous decline corresponding to displacement increments. During the later loading stage with larger displacement, the stiffness degradation demonstrated a more gradual progression compared to the initial loading phase, attributable to the yielding of the internal steel reinforcement.

(2) Comparing different mold shell materials (E2S38R, R2S38R), the initial stiffness of specimen R2S38R was greater than that of the ECC composite beam specimen, which was because the ordinary concrete in the RC composite beam mold shell material contained coarse aggregate. The yield loads of specimen E2S38R and specimen R2S38R were 358.57 kN and 346.24 kN, respectively. From the stiffness-displacement curve after the yield load, it could be concluded that the stiffness degradation slope of specimen R2S38R was −4.34, and the stiffness degradation slope of specimen E2S38R was −1.58. The stiffness degradation rate of specimen R2S38R after yield was significantly greater than that of specimen E2S38R. This was because after the specimen entered the yield stage, the ordinary concrete in the mold shell of specimen R2S38R was continuously peeled off, resulting in a smaller cross-sectional area of the effective bearing capacity of the RC composite beam, which resulted in faster stiffness degradation. The ECC material in the shell of specimen E2S38R formed multiple fine cracks by fiber bridging. After cracking, it was forced together with the steel bar, and there was no spalling. Therefore, the stiffness degradation of specimen E2S38R was more gentle than that of specimen R2S38R. It also showed that the ECC shell material could effectively slow down the stiffness degradation rate of the composite beam.

(3) Comparing different shear span ratios, the shear span ratios of specimens E3S38R, E2S38R, and E1.5S38R were 3, 2, and 1.5, respectively. The initial stiffness of the specimens was 62.42 kN/mm, 71.11 kN/mm, and 80.26 kN/mm, respectively, indicating that under the same other parameters, the larger the shear span ratio of the specimen, the smaller the initial stiffness. When the specimen yielded, the stiffness of the specimen was 23.23 kN/mm, 30.57 kN/mm, and 35.93 kN/mm, respectively. With the increase in load, specimen E1.5S38R experienced baroclinic failure, showing brittle failure, which resulted in a sharp decrease in stiffness, while the stiffness degradation of specimens E3S38R and E2S38R was relatively slow.

(4) Comparing different stirrup ratios, the stirrup ratios of specimens E2S25R, E2S38R, and E2S75R were 0.25%, 0.38%, and 0.75%, respectively. The initial stiffness of E2S75R (74.50 kN/mm) was higher than that of E2S38R (71.11 kN/mm) and E2S25R (69.38 kN/mm) by 4.53% and 6.86%, respectively. The reason was that the stirrup ratio of E2S75R was 49.33% and 66.67% higher than that of E2S38R and E2S25R, respectively. When specimens E2S25R, E2S38R, and E2S75R reached the yield load, the stiffness of each load level decreased, as shown in Table 7:

It can be seen from Table 7 that under each test load, the higher the stirrup ratio of the specimen, the smaller the stiffness degradation of the specimen.

### 3.6. Strain Analysis of Longitudinal Reinforcement

**Figure 15:**
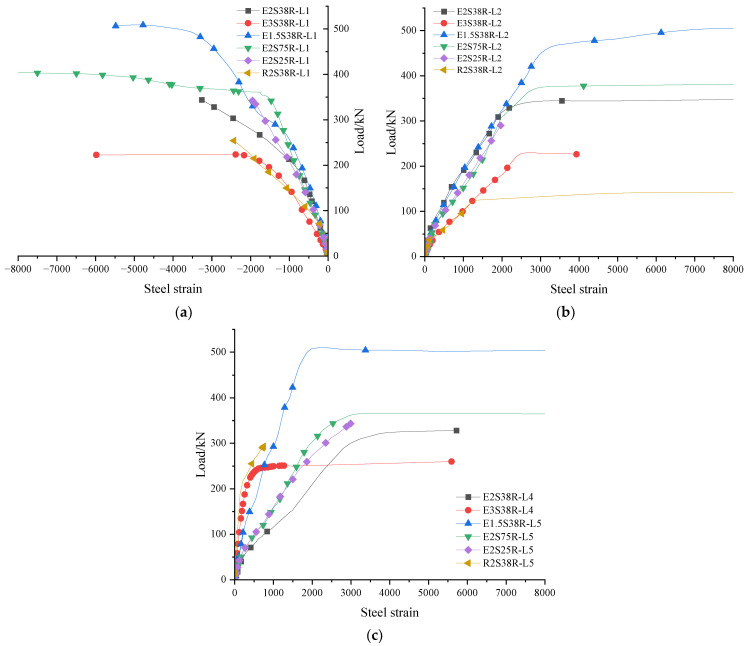
Load–steel strain diagram (**a**) Upper longitudinal reinforcement; (**b**) lower longitudinal reinforcement; (**c**) the longitudinal reinforcement in the mold shell.

The longitudinal reinforcement strain gauge was numbered as L series, and the layout is shown in Figure 5. The load–steel strain diagram drawn from the data measured by the experiment is shown in Figure 15, and the data of each strain gauge rose steadily, indicating that there was no relative slip between the steel bar and the concrete and ECC mold shell.

(1) From Figure 15a, it can be seen that compared with different mold shell materials (E2S38R and R2S38R), the load of the first vertical crack of the R2S38R specimen (78 kN) was 35.5% smaller than the load of specimen E2S38R (121 kN), resulting in the upper longitudinal reinforcement strain of the R2S38R specimen being greater than that of specimen E2S38R at the same load level. When the load was 100 kN, the strain of the R2S38R specimen (−459.6 με) was 28.9% higher than that of specimen E2S38R (326.9 με). At 200 kN, the strain of the R2S38R specimen (1793.4 με) was 51.33% higher than that of the E2S38R specimen (872.7 με). It was shown that the RC shell was more likely to crack under low load than the ECC shell, and the crack released the stress in advance, forcing the upper longitudinal reinforcement to bear more tensile stress. Comparing the different shear span ratios of the specimens, the corresponding strains of specimens E3S38R, E2S38R, and E1.5S38R with shear span ratios of 3, 2, and 1.5 were 663.5 με, 337.9 με, and 266.8 με, respectively, when the load was 100 kN, and the decreases were 49.1% and 21.0%, respectively. When the load was 200 kN, the corresponding strains of specimens E3S38R, E2S38R, and E1.5S38R were 1588.2 με, 896.0 με, and 722.3 με, respectively, and the decreases were 43.6% and 19.4%, respectively. It showed that the strain of the compressive longitudinal reinforcement in the upper part of the specimen decreased with the decrease in the shear span ratio of the specimen. The high shear span ratio (E3S38R) was dominated by bending deformation, the strain in the tensile zone of concrete was significant, the cracks developed along the mid-span, and the strain accumulation rate of longitudinal reinforcement was fast. The shear effect of the low shear span ratio (E1.5S38R) was enhanced, the main oblique cracks were rapidly penetrated, the compressive strain of concrete was dominant, and the strain growth rate of longitudinal reinforcement was slowed down.

(2) It can be seen from Figure 15b that, compared with different mold shell materials (E2S38R and R2S38R), the load corresponding to the yield of the lower longitudinal reinforcement of the specimen R2S38R was 125 kN, while the load corresponding to the yield of the lower longitudinal reinforcement of the specimen E2S38R was 322.6 kN, which was 61.25% higher than that of the R2S38R specimen. It showed that ECC had strain-hardening characteristics and could form dense microcracks when subjected to tension. This multi-crack cracking mechanism effectively dispersed the stress concentration and delayed the formation of main cracks, thus protecting the longitudinal reinforcement from premature yield.

A comparison of specimens with varying shear span ratios (λ = 3 for E3S38R, λ = 2 for E2S38R, and λ = 1.5 for E1.5S38R) revealed distinct strain patterns in the lower longitudinal reinforcement. At 100 kN loading, the measured strains were 946.2 με (E3S38R), 358.1 με (E2S38R), and 403.5 με (E1.5S38R). This differential became more pronounced at 200 kN, with strain values increasing to 2172.8 με (E3S38R), 1092.1 με (E2S38R), and 1041.9 με (E1.5S38R). Notably, at 300 kN loading, E2S38R and E1.5S38R specimens reached similar strain levels of 1828 με and 1812.6 με, respectively. The strain development patterns demonstrated two critical observations: (1) E2S38R and E3S38R specimens shared comparable ascending phases in their strain curves, despite their different λ values. (2) Specimens with λ = 3 consistently exhibited 33–121% higher reinforcement strains than those with λ = 2 and 1.5 across loading stages. This strain disparity was directly correlated with bending moment magnitude in the mid-span region, where increased shear span ratios (λ) amplified flexural effects, thereby elevating tensile strain in the lower longitudinal reinforcement.

(3) It can be seen from Figure 15c that, compared with different mold shell materials (E2S38R and R2S38R), the strain of the longitudinal reinforcement in the mold shell of specimen R2S38R began to increase from 180 kN, while the lower longitudinal reinforcement of specimen R2S38R had already yielded at 127 kN. With the yield of the lower longitudinal reinforcement, when the load reached 300 kN, the strain of the longitudinal reinforcement in the mold shell suddenly increased to 752 με, and then the strain gauge failed. When the load was less than 127 kN, the lower longitudinal reinforcement dominated the bending resistance, and the bond-slip at the die shell interface accumulated. When the load exceeded 127 kN, after the lower longitudinal reinforcement yielded, the longitudinal reinforcement in the shell was forced to bear the bending moment increment. However, due to insufficient interface constraints, the strain concentration accelerated local failure.

### 3.7. Stirrup Strain Analysis

**Figure 16:**
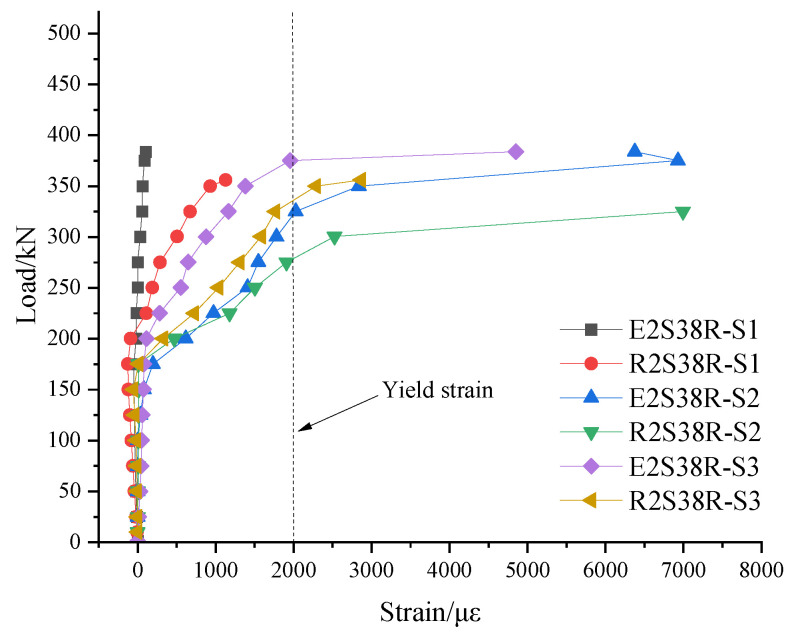
Load–stirrup strain curve of the shell material control group.

**Figure 17:**
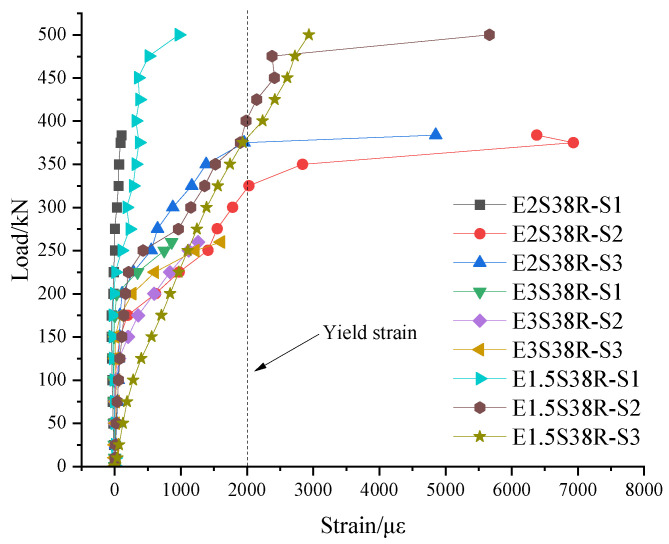
Shear span ratio control group load–stirrup strain curve.

S1 through S6 were the designations for the strain gauges. S1 to S3 were installed along the line connecting the support and the loading point, while S4 to S6 were installed at symmetrically opposite locations on the back face, corresponding to S1–S3. The specific arrangement is shown in Figure 5. In the same control group of specimens, the stirrup strain gauges at the corresponding positions were selected for comparative analysis. The specific analysis process was as follows:

(1) Comparing the specimens of different shell materials, the load–stirrup strain of specimens E2S38R and R2S38R is shown in Figure 16. It could be seen from the figure that at the S1 position, the strain of the stirrup was close to a linear change before the oblique crack appeared. When the R2S38R load of the specimen reached 170.3 kN, the lower longitudinal reinforcement yielded. At this time, the stirrup at the S1 position bore more shear force, which made the strain increase rapidly. After the crack of specimen E2S38R appeared, the tensile force was borne by the fiber between the cracks and the lower longitudinal reinforcement, the yield of the longitudinal reinforcement was relatively late (307 kN), and there was no oblique crack at the S1 position, resulting in little change in the stirrup at the S1 position. At the S2 position, oblique cracks appeared when R2S38R was 170 kN, which made the stirrup strain at the S2 position increase sharply. At the S2 and S3 positions, the stirrup strain changes of specimens E2S38R and R2S38R were roughly the same. Under the same load, the stirrup strain value of the E2S38R specimen was less than that of R2S38R, which indicated that the characteristics of the fiber material in the ECC shell could provide partial shear resistance.

(2) The shear span ratios of specimens E3S38R, E2S38R, and E1.5S38R were 3, 2, and 1.5, respectively. The stirrup load–strain curve of the shear span ratio control group is shown in Figure 17. When the strain increased to 908 με, the strain gauge was destroyed, and the unit reached the yield strain of the steel bar. It showed that when the stirrup in the S1 position had not yet yielded, the bond between the stirrup and the concrete was destroyed, resulting in the damage of the stirrup strain gauge; the stirrup strain at the S1 position of the E2S38R specimen did not change during the loading cycle; the strain gauge at the S1 position of the E1.5S38R specimen changed to 0 before the load reached 226.5 kN, and increased rapidly from 226.5 kN to 449 kN. Similar to specimen E3S38R, when the stirrup had not yet yielded, the bond between the stirrup and the concrete was destroyed, resulting in damage to the stirrup strain gauge. It was shown that when the shear span ratio was 3 (E3S38R) and 1.5 (E1.5S38R), the bond between the stirrup and the concrete near the bearing end of the test beam was destroyed in the later stage of loading, while when the shear span ratio was 2, it did not occur.

In the S2 position, the strain of the E3S38R specimen increased suddenly at 100 kN, and the bond between the steel bar and the concrete was destroyed when the strain increased to 1263 με, resulting in the damage of the strain gauge; the strain gauges of specimen E2S38R and specimen E1.5S38R increased steadily at 173 kN. The stirrup of the E2S38R specimen yielded when the load was 326 kN. When the load reached the yield load of 383.97 kN, the stirrup strain was 6350 με, indicating that the strengthening stage after the stirrup yield was fully exerted and the ductility of the component was enhanced. The E1.5S38R stirrup yielded 403 kN. Similar to specimen E2S38R, the strengthening stage of the stirrups was fully utilized.

At the S3 position, the specimen E1.5S38R with a shear span ratio of 3 began to grow at the S3 position of 145 kN. When the strain increased to 1596 με, the bond between the stirrup and the concrete was destroyed, failing the strain gauge. The shear span ratio of specimen E2S38R was 2. When the load exceeded 198.9 kN, it grew steadily and slowly. When the load reached 375 kN, the stirrup strain at the S3 position reached 2015 με, and the maximum strain after yielding was 4860 με. Until the end of the test, the strain gauge did not fail due to damage, indicating that the bond between the stirrup and concrete was good. In this comparison group, the shear span ratio of E1.5S38R was the smallest at 1.5. Due to the small shear span ratio, the stirrup at the S3 position was forced from the beginning of the test, and the overall relationship was linear. When the load was 381 kN, the stirrup strain reached 2025 με and yielded. After yielding, the stirrup continued to grow until the end of the test.

**Figure 18:**
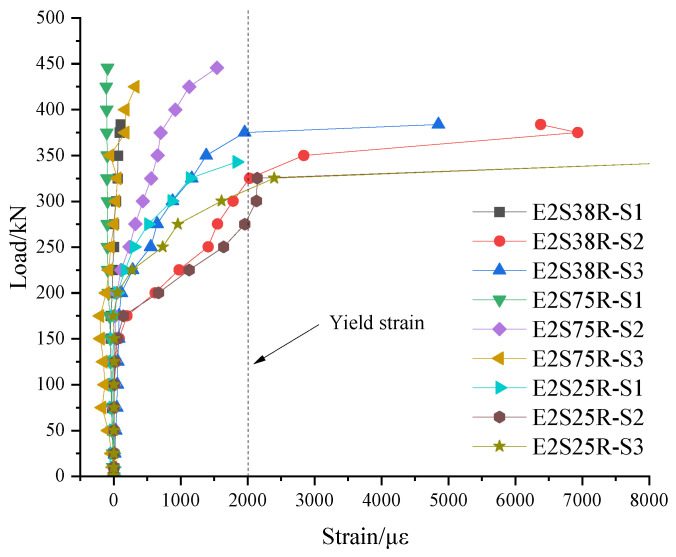
Stirrup ratio control group load–stirrup strain curve.

(3) Effect of stirrup ratio: The stirrup ratios of specimens E2S25R, E2S38R, and E2S75R were 0.25%, 0.38% and 0.75%. The load–stirrup strain diagram of the comparison group is shown in Figure 18.

At the S1 position, the stirrup strain of this group of specimens was quite different. The stirrup strain of specimens E2S38R and E2S75R at the S1 position did not change during the whole test. E2S25R with a stirrup ratio of 0.25% increased when the load reached 172 kN. When the load reached the ultimate load of the specimen of 344.7 kN, the corresponding stirrup strain was 1879 με, indicating that the stirrup did not yield before the failure of the component, showing brittle failure. When the load was 250 kN, the stirrup strains of E2S25R, E2S38R, and E2S75R were 294.6 με, 19.4 με, and 12.1 με; when the load was 325 kN, the stirrup strains of E2S25R, E2S38R, and E2S75R were 1143.4 με, 40.7 με, and 26.6 με, respectively.

In the S2 position, the stirrup strain of this group of specimens before 153 kN did not show a significant change. Because the maximum stirrup ratio of specimen E2S75R was 0.75% when the specimen reached the ultimate load of 444.07 kN, the stirrup strain was 1548 με and did not yield, and the strength of the stirrup was not fully exerted. The specimen E2S25R was roughly the same as the specimen E2S38R. The stirrup strain did not change much before 126 kN. When the load reached 274 kN, the stirrup strain of the E2S38R specimen was 1544 με, and the E2S25R specimen was 1976 με. Under this load, the stirrup strain of E2S25R was 27.8% higher than that of the E2S38R specimen. When the stirrup reached the yield strain at the S2 position, the load of the E2S38R specimen was 15.3% higher than that of E2S25R. When the load was 200 kN, the stirrup strains of E2S25R, E2S38R, and E2S75R were 715 με, 612 με, and 34.9 με, respectively. When the load was 300 kN, the stirrup strains of E2S25R, E2S38R, and E2S75R were 2133.7 με, 1775.3 με, and 424.4 με, respectively.

At the S3 position, the strain of the specimen E2S75R did not change much. When the load was 425 kN, the stirrup strain was 284.9 με, and the stirrup at the S3 position of the specimen did not yield. When the specimens E2S38R and E2S25R were at 200 kN, the stirrup strain rose steadily until it yielded. When the load was 250 kN, the stirrup strains of E2S25R, E2S38R, and E2S75R were 748 με, 534 με, and −17.4 με, respectively. When the load was 300 kN, the stirrup strains of E2S25R, E2S38R, and E2S75R were 1606.6 με, 862.4 με, and 44 με, respectively.

The comprehensive analysis showed that the strain of the stirrup decreased with the increase in the stirrup ratio under the same level of load. The specimen E2S25R (stirrup ratio was 0.25%) with the lowest reinforcement ratio was close to yield in the stirrups at three positions, and the strength and ductility of the stirrups were fully utilized, but it presented brittle failure. For specimen E2S38R with a stirrup ratio of 0.38%, except that the stirrups at the S1 position were not yielded, the stirrups at the other positions were yielded, and the strain could continue to develop after yielding. Due to the high reinforcement ratio of specimen E2S75R with a stirrup ratio of 0.75%, the stirrups at the three positions did not yield, and the maximum stirrup strain was 1520 με, indicating that the stress utilization rate of the stirrups was not high, and their strength and ductility were not fully utilized.

## 4. Conclusions

This study experimentally investigates the shear behavior of five ECC composite precast beams and one RC composite beam, focusing on the effects of formwork materials, shear-span ratios, and stirrup ratios. The key findings are summarized as follows:

- The precast monolithic ECC composite beams showed superior integrity and shear performance. The ECC composite beams limited maximum diagonal crack width to 2.2 mm (76.1% reduction compared to RC beams’ 9.2 mm), exhibiting fine, distributed microcracks without concrete spalling. The ECC composite beam exhibited an 8.2% higher ultimate load-bearing capacity compared to the conventional concrete composite beam, with a 29.3% improvement in the corresponding displacement.
- The ECC composite beam demonstrated superior performance with an average degradation coefficient of 0.067 (8.2% lower than the RC beam), confirming ECC’s effectiveness in delaying strength deterioration. At the post-yield stage, the stiffness degradation slope was much slower than that of the RC beam, validating enhanced ductility.
- The ECC composite beams exhibited consistent mechanical performance within the shear span ratio (λ) range of 1.5–3.0. Increasing the ratio (λ) from 1.5 to 3.0 resulted in a 24.4–32.0% reduction in shear capacity, accompanied by a transition in failure pattern from diagonal compression failure (λ = 1.5, ultimate load: 507.92 kN) to flexural failure (λ = 3.0, ultimate load: 261.09 kN).
- The ECC composite beams maintained satisfactory shear performance even at a stirrup ratio of 0.25%. Raising the stirrup ratio from 0.25% to 0.75% enhanced the ultimate capacity by 28.8%. The stiffness degradation of specimen E2S75R was 40–50% less than that of specimen E2S25R.

The results provided theoretical foundations and technical support for enhancing the mechanical performance of assembled monolithic structures and promoting their engineering applications. However, this study was limited to one RC reference specimen and only investigated the effects of shear span ratio and stirrup ratio on the shear behavior of ECC composite beams. The additional control specimens should be investigated, and other critical factors, including ECC material strength and shell thickness, will be studied in further research to enhance the reliability of conclusions.

## Figures and Tables

**Table 1:** Main parameters of composite beam specimens.

Specimen ID	Shell Material	Shear Span Ratio	*L*_0_ /mm	Longitudinal Reinforcement of Mold Shell	Top Longitudinal Reinforcement	Bottom Longitudinal Reinforcement	Hooped Tendon	Stirrup Ratio /*ρ_sv_*
E2S38R	ECC	2	1200	2C18	2C16	2C12	A6@100	0.38%
E3S38R	ECC	3	1800	2C18	2C16	2C12	A6@100	0.38%
E1.5S38R	ECC	1.5	900	2C18	2C16	2C12	A6@100	0.38%
E2S75R	ECC	2	1200	2C18	2C16	2C12	A6@50	0.75%
E2S25R	ECC	2	1200	2C18	2C16	2C12	A6@150	0.25%
R2S38R	RC	2	1200	2C18	2C16	2C12	A6@100	0.38%

Note: The letter E denotes that the shell material was engineered cementitious composite (ECC), while R indicates conventional concrete. The notation 2S signifies a shear span ratio of 2 (other shear span ratios followed the same convention). The 75R corresponds to a longitudinal reinforcement ratio of 0.75% (other reinforcement ratios were similarly encoded). *L*_0_ represents the center-to-center distance between the specimen’s loading supports. The stirrup ratio was defined as the ratio of the volume or cross-sectional area of stirrups to the volume or cross-sectional area of the confined concrete core. The shear span ratio (λ) is defined as *λ* = *a*/*h*_0_.

**Table 2:** PVA fiber performance indicators.

Fiber Name	Length /mm	Diameter /μm	Tensile Strength /Mpa	Elastic Modulus /GPa	Elongation /%
PVA	12	40	1560	41	6.5

**Table 3:** ECC mix proportion (kg/m^3^).

Cement	Fly Ash	Water	Quartz Sand	Superplasticizer	Polyvinyl Alcohol
659.71	659.71	445	480.59	16	26

**Table 4:** Mechanical properties of concrete.

Item	Material	Dimensions of Reserved Specimens /mm	Average Test Value /MPa
Cube crushing strength	Concrete	150 × 150 × 150	47.8
	ECC	100 × 100 × 100	50.0
Axial compressive strength	Concrete	150 × 150 × 300	42.8
	ECC	100 × 100 × 300	43.2
Tensile strength	ECC	Dog-bone specimen	4.16

**Table 5:** Characteristic load values and failure patterns of test beams.

Specimen ID	Shear Span Ratio	Vertical Crack Initiation Load /kN	Diagonal Crack Initiation Load /kN	Yield Load /kN	Displacement at Yield Load /mm	Ultimate Load /kN	Displacement at Ultimate Load /mm	Failure Pattern
E2S38R	2.0	121	121	358.57	11.73	383.97	17.48	Shear-Compression Failure
E3S38R	3.0	83.54	207	167.93	7.23	261.09	48.64	Flexural Failure
E1.5S38R	1.5	175.9	227.8	503	14.00	507.92	14.62	Shear-Compression Failure
E2S75R	2.0	114	188	396.58	20.51	444.07	37.66	Shear-Compression Failure
E2S25R	2.0	162	276	265	8.42	344.70	10.33	Shear-Compression Failure
R2S38R	2.0	78	170	346.24	11.10	352.13	12.36	Shear-Compression Failure

**Table 6:** Results of bearing capacity degradation coefficient.

Specimen ID	λ1	λ2	λ3	λ4	Average λ
E2S38R	0.017	0.087	0.101	0.063	0.067
E3S38R	0.040	0.026	0.021	0.012	0.025
E1.5S38R	0.156	0.122	0.106	0.090	0.118
E2S75R	0.006	0.055	0.067	0.123	0.063
E2S25R	0.229	0.174	0.146	0.114	0.166
R2S38R	0.049	0.114	0.052	0.076	0.073

**Table 7:** Stiffness degradation range table.

Load Series	E2S25R	E2S38R	E2S75R
Level 1	10.04%	7.2%	5.43%
Level 2	17.17%	10.86%	10.11%
Level 3	21.31%	13.13%	10.95%

## Data Availability

The original contributions presented in the study are included in the article. Further inquiries can be directed to the corresponding author.
